# Effects of rice-based and wheat-based diets on bowel movements in young Korean women with functional constipation

**DOI:** 10.1038/s41430-020-0636-1

**Published:** 2020-04-22

**Authors:** Su-Jin Jung, Mi-Ra Oh, Soo-Hyun Park, Soo-Wan Chae

**Affiliations:** 1 Clinical Trial Center for Functional Foods, Chonbuk National University Hospital, Jeonju, Jeonbuk 54907 Republic of Korea; 2 Biomedical Research Institute, Chonbuk National University Hospital, Jeonju, Jeonbuk 54907 Republic of Korea; 3 Department of Pharmacology, Jeonbuk National University Medical School, Jeonju, Jeonbuk 54896 Republic of Korea

**Keywords:** Gastroenteritis, Nutrition

## Abstract

**Background:**

Although several studies have reported the effects that dietary fiber intake from different types of grains and fiber components have on bowel movements, insufficient attention has been paid to comparing and evaluating the effects of rice-based and wheat-based diets. This study compared and evaluated the effects of ingesting rice-based (brown rice-based diet: BRD; white rice-based diet: WRD) and wheat-based diet (WD) on the bowel movements of young women with functional constipation.

**Method:**

Based on an open, randomized, controlled, and parallel design, 39 subjects were assigned to BRD, WRD, and WD groups (13 in each group). Each participant had received three types of experimental diets over the course of 4 weeks and we recommended that the subjects eat only the test diet provided during the study. Primary outcomes (total colon transit time TCTT) and secondary outcomes (bowel movements, short-chain fatty acid content, and fecal enzyme activity) were compared before and after the 4-week intervention period.

**Results:**

After the 4-week study, the rice-based diet (BRD and WRD) groups and the WD group had a statistically significant difference in TCTT (*p* = 0.028). The TCTT of the BRD group was significantly reduced (*p* = 0.028) compared with the WRD group (−16.5 ± 8.1 vs +6.8 ± 2.1), and the TCTT of the WD group was also significantly reduced (*p* = 0.022) compared with that of the WRD group (−17.1 ± 11.9 vs +6.8 ± 2.1).

**Conclusion:**

Among women with functional constipation, the BRD and WD both improved bowel function by reducing TCTT and increasing the number of bowel movements compared with the WRD group.

## Introduction

Constipation is a common digestive disorder, and insufficient dietary fiber intake is one of its major causes. In chronic constipation patients, dietary fiber promotes colonic mobility and improves bowel movements, which reduces the time that carcinogens remain in the body and thereby suppresses the outbreak of colon cancer [1]. However, although increasing dietary fiber intake can be an efficient way to treat mild and moderate chronic constipation, it is not always effective in cases of severe constipation [2]. It is generally recommended that individuals consume 20–25 g of dietary fiber per day to improve constipation. The daily recommended dietary fiber intake for Koreans is 12 g/1000 kcal, but the actual average intake is only half that [3–5]. In general, fiber supplements are recommended to increase the fiber consumption of constipation patients. However, recent clinical studies have reported that high fiber diets, especially those in which the fiber is consumed as supplements, can cause intestinal discomfort such as convulsive abdominal pain and gas production, and even constipation or fecal impaction [6–9]. In addition, several systematic reviews and meta analyses have reported that although dietary fiber increased the number of bowel movements in constipation patients, it was unclear whether it improved fecal consistency [6, 8, 9]. Previous reports have discussed the relevance of fiber supplements to complement a lack of natural dietary fiber intake, such as resistant maltodextrin, beta-glucan, psyllium, inulin, cellulose, and konjac glucomannan, to bowel movements [7, 9–15]. It is important to find a safe and cost-effective way for constipation patients to consume adequate dietary fiber that minimizes side effects while relieving and preventing constipation. The most common food group consumed by humans per day is grains, which are divided into two categories, whole grains and refined grains. The whole grains include brown rice, whole wheat, oatmeal, rye, barley, corn, popcorn, buckwheat, quinoa, and sorghum, and the refined grains include refined flour or white rice from which the bran and germ have been removed. Most whole grains are a good source of dietary fiber, but refined grains contain little fiber. Moreover, whole grains improve bowel movements, increase fecal weight, soften feces, and help to normalize bowel movements and improve constipation by reducing colonic transit time (CCT) [4, 14–19]. Studies of the relationship between the dietary fiber of grains and bowel movements have frequently been reported, whereas studies comparing various cereal dietary fibers with wheat fiber are insufficient [16, 20, 21]. A few correlation studies [22] reported bowel movements according to the intake of single or mixed grains, whole meal rye bread [23], and vegetables and whole-grain powder [24]. Studies comparing and evaluating the effects of a rice-based diet and a wheat-based diet on bowel movements are insufficient. In summary, efficient measures are needed to help individuals consume enough dietary fiber from natural sources in daily life to improve their bowel movements, rather than to consume fiber supplements separated or synthesized from a particular natural food.

In this study, we compare and evaluate the effects and safety of eating rice-based diets (brown rice-based and white rice-based) and a wheat-based diet in terms of bowel health and bowel movements among young women with functional constipation.

## Subjects and methods

### Subjects

This study was conducted with the review and approval (CBNUH_CTCF2_IRB 2012–01–002) of the Institutional Review Board of Chonbuk National University Hospital. The study was conducted according to the Helsinki Declaration and the Guideline for Good Clinical Practice by the International Conference on Harmonization. This study protocol was registered at www.clinicaltrials.gov (NCT01933100), and the dietary intervention was carried out for four weeks from September 16 to December 12, 2012. A total of 39 participants who met the selection criteria were chosen by applying a screening test to 46 volunteers who prepared written consent.

#### Selection criteria

The women included in this study were 19–30 years old and met the diagnostic criteria for Rome III functional constipation (had two or more of the six items in the Rome III functional constipation diagnosis criteria, but did not meet the criteria for hypersensitivity bowel syndrome) [25] without any loose feces or watery diarrhea in the past three months. They also received and understood a full explanation of this human study and agreed in writing to participate and to comply with all precautions.

#### Exclusion criteria

The following subjects were excluded from this study: women with diseases of the digestive system, anorectal system, liver, kidney, nervous system, respiratory system, endocrine system, cardiovascular system, or blood or with a tumor, mental illness, or past disease history; those with a past history of gastrointestinal diseases (e.g., Crohn’s disease) or gastrointestinal surgery (except for simple appendectomy or hernia) that could affect the absorption of the diet applied in this human study; those whose AST or ALT were greater than twice the upper limit; those with a history of hypersensitive reactions or clinically significant hypersensitivity to white rice, brown rice, wheat, milk, or other foods; those who continued to eat foods containing lactobacillus (milk, yogurt, etc.); those who took drugs that affect digestive canal movements within two weeks of the first day of intake (unless other conditions were reasonable in the opinion of the test manager); those who participated in other human studies within two months of the screening test; those who drank 21 units/week or more of alcohol; those who were pregnant or nursing; those who were fertile and not taking contraceptives; those who met the Rome III diagnostic criteria for hyperactive growth syndrome (two or more of the following three categories: having felt pain or discomfort anywhere in the abdomen during the past three months that improved or vanished after bowel movements, began at the same time as changes in the number of bowel movements, or began at the same time as the fecal consistency changed); and those deemed by the test manager to be unfit for participation for any other reason, including the results of laboratory testing.

### Study design

This dietary intervention study used an open, randomized, parallel-group comparison method. The subjects were assigned 1:1:1 to the brown rice-based diet (BRD) group (*n* = 13), WRD group (*n* = 13), or WD group (*n* = 13) through a random assignment method. All subjects assigned to each diet group visited the clinical research center every day for four weeks and ate meals provided by the researchers at 7 a.m. (breakfast), 12 p.m. (lunch), and 6 p.m. (dinner). The test diets of this study consisted of a 14-day menu cycle (Supplementary Fig. 1). Considering the characteristics of the subjects who participated in this study, an estimated daily energy requirement of 2100 kcal (carbohydrate: 55–70%, protein: 7–20%, lipid: 15–25%) was provided, and the recommended daily intake rate for each diet group was applied (Dietary reference intakes for Koreans, 2010) [26]. The average dietary fiber intake for each group was 30 g/day, which is higher than the sufficient recommended intake of 20 g/d, to improve the subjects’ bowel movements. The average daily energy and nutrients provided to the study subjects are presented in Table 1.

**Table 1:** Mean nutrient contents provided in each intervention diet group per day.

Nutrients	BRD	WRD	WD
Energy (kcal)	2,100	2,100	2,100
Carbohydrate (g)	314	314	340
Carbohydrate (%)	60	60	61
Protein (g)	108	108	78
Protein (%)	20	20	15
Lipids (g)	57	57	59
Lipids (%)	20	20	24
Cholesterol (mg)	444	444	404
Total fatty acids (g)	36.1	36.1	30.2
Saturated fatty acids (g)	12.6	12.7	9
Mono-saturated fatty acids (g)	18.2	18.2	10.1
Unsaturated fatty acids (g)	15.8	15.8	10.2
Fiber (g)	39	34	34

*BRD* brown rice-based diet, *WRD* white rice-based diet, *WD* wheat-based diet.

#### Brown rice-based diet and white rice-based diet

The dietary compositions of the BRD and WRD groups were provided by applying the same daily calorie content and ratio of carbohydrates, proteins, and fats. The difference between these two diets was that the BRD group ate a 100% whole grain (brown rice), and the WRD group ate the same quantity of white rice. All the other foods (soups, side dishes) were provided equally. To reflect the characteristics of rice-based diets, the staple and subsidiary foods were provided in strictly separate forms. Also, milk and dairy products were excluded from the test diet, except for bread and noodles, which are flour-based foods.

#### Wheat-based diet

The WD group was fed a ratio of calories and nutrients similar to that of the rice-based (BRD and WRD) groups [26]. The diet of the WD group contained grains such as breads, soups, noodles, cereals, dumplings, and sandwiches to reflect the characteristics of wheat-based meals as much as possible. The subsidiary foods were salads, pickles, nuts, milk and dairy products, and fruit and vegetable juices.

#### Subject compliance

To minimize the effects of lifestyle changes on the test results, we recommended that the subjects eat only the test diet provided during the study and that they maintain their physical activity levels from before the study. We evaluated dietary compliance and physical activity during the study. The subjects recorded their daily diet intake in detail on a dietary record and were monitored for drug use and self-reported symptoms or side effects, changes in physical activity, lifestyle habits, and the suitability of their diet.

### Outcome measures

All subjects received efficacy and safety evaluations before and after their participation in this four-week study. The primary outcome was total colonic transit time (TCTT). The secondary outcomes were the number of bowel movements, fecal weight, fecal short-chain fatty acid content, and fecal enzyme activity before and after participation. The safety measures were adverse events, diagnostic tests, vital signs, physical examinations, and electrocardiogram tests.

#### Primary outcomes

##### Total colonic transit time

The primary outcome was the TCTT, which was measured in all subjects in the first visit (Week 0) and second visit (Week 4) of the intervention period using the CTT method described by Metcalf et al. [27]. The subjects of this study took one capsule, which contains 20 radiopaque markers, for three days for the first visit (Days −3 - −1) and the second visit (Days 26–28). Then, an abdominal X-ray was taken to identify the number of markers remaining in the colon in their first and second visits. CTT was calculated as the sum of the markers detected on X-ray. The sum of the markers detected in the X-ray was multiplied by 1.2, resulting in CTT expressed in hours.

The measurement of the CTT was performed and evaluated using radio-opaque markers. Plain abdominal roentgenograms were read in three segments: right colon, left colon, and recto-sigmoid, using the bone structure of the spine and pelvis and the intestinal air shading (Supplementary Fig. 2). In general, the method of colonic segmentation is to designate the right side of the line that connects the spinous processes of the spine and the right side of the line that connects the pelvic outlet in the 5th lumbar spine body as the right colon. The upper part of the line connecting the anterior superior iliac spine on the left side of the spinous processes and the 5th lumbar spine body was designated as the left colon. In the 5th lumbar spine body, below the pelvic outlet extension line on the right and below the anterior superior iliac spine extension line on the left, is read as the recto-sigmoid [28]. In this study, the CTT measurements and readings were implemented according to the identification codes of the subjects assigned by the expression (Block Random Identification Code: Subject ID), and the blinding was maintained until the study was completed.

#### Secondary outcomes

##### Fecal sample collection and analysis

The secondary outcome measures were: (1) number of bowel movements; (2) fecal weight (g/d); (3) short-chain fatty acid content in the feces; and (4) fecal enzyme activity. The subjects were asked to keep a bowel movement diary every day for 4 weeks. The diary included defecation frequency, bowel time, degree of difficulty in defecation, and abdominal symptoms. At the beginning and end of the trial, the participants were asked to measure the weight of a whole stool and to bring a small amount of frozen stool to the study site in a plastic bag for a biochemical analysis of fecal short-chain fatty acids (lactic acid, butyric acid, and propionic acid), and urease, β-glucosidase, and β-glucuronidase activity.

##### Dietary intake investigation

The dietary intake investigation in this study was explained to the subjects by a trained nutritionist, who also gave instructions for preparing a diet record. To check the dietary intake of all the subjects, the amount they left uneaten during the four-week period was measured. All the subjects were instructed to consume only the test diet provided and to record any additional food items consumed in the diet record so they could be reflected in the analysis. The dietary intake analysis was performed using Can-pro 4.0 (computer-aided nutritional analysis program, The Korean Nutrition Society Forum, Seoul, Korea) with the data from the 28 days recorded during the four-week study from which the average daily calorie and nutrient intake were calculated.

##### Compliance and safety testing of the test diets

We calculated the compliance of all study subjects by subtracting the number of meals they consumed per day from the total number offered. Subjects whose compliance did not exceed 70% were excluded from the analysis.

(Example):

$${\mathrm{Diet}}\,{\mathrm{compliance}}\left( \% \right) = \frac{{{\mathrm{the}}\,{\mathrm{number}}\,{\mathrm{of}}\,{\mathrm{meals}}\,{\mathrm{in}}\,{\mathrm{four}}\,{\mathrm{weeks}}-{\mathrm{the}}\,{\mathrm{number}}\,{\mathrm{of}}\,{\mathrm{uneaten}}\,{\mathrm{meals}}}}{{{\mathrm{the}}\,{\mathrm{number}}\,{\mathrm{of}}\,{\mathrm{meals}}\,{\mathrm{in}}\,{\mathrm{four}}\,{\mathrm{weeks}}}} \times 100$$

### Statistical analysis

All statistical analyses were implemented using SAS 9.2 (Statistical Analysis System version 9.2, SAS Institute, Cary, NC, USA) and SPSS 20 (IBM Co., Armonk, NY, USA). Continuous variables are presented as the mean ± standard deviation (SD), and categorical variables are presented as frequencies. Efficacy and safety parameters were analyzed within the intention-to-treat (ITT) group. The homogeneity test and baseline homogeneity test between the groups were conducted using the chi-square test and Wilcoxon rank-sum test. The validity evaluation items were analyzed using a linear mixed-effect model with the Bonferroni test to compare differences among the diet groups for CTT, bowel movements, fecal weight, pH, short-chain fatty acid content in the feces, and fecal enzyme activity. Changes in the results within each test group before and after the four-week study were compared and evaluated using paired t-tests. During the dietary intervention period, the nutrient intake in each group was analyzed using analysis of variance for comparative evaluation, and the Bonferroni correction multiple range post hoc test was used. All statistical significance levels were set at *p* < 0.05.

### Sample size

This pilot study was the first clinical trial on rice-based diets in young Korean women, so limited information was available beforehand about functional constipation in that population. Therefore, we designed this preliminary test for 39 subjects.

### Adverse events and safety monitoring

The blood testing done in this study measured WBC, RBC, hemoglobin, hematocrit, platelets, and the activity of γ-GT, AST, and ALT, which indicate liver function. Indicators of kidney function (total bilirubin, total protein, BUN, and creatine kinase) were measured using a colorimetric method with a Hitachi 7600–110 (Hitachi, Tokyo, Japan). Albumin, total cholesterol, triglyceride, glucose, and urine tests were also performed. The blood and urine tests were conducted before the study (week 0) and four weeks later. We trained the subjects to voluntarily report any adverse events. The vital signs test measured systolic blood pressure, diastolic blood pressure, and pulse at each visit.

## Results

### Baseline demographics

**Fig. 1:**
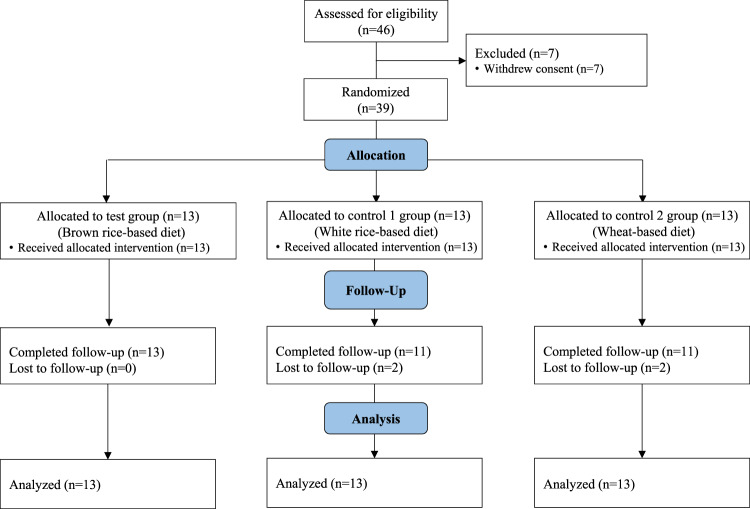
Flow chart of the participants in this study.

Originally, 39 subjects participated in the study, and 13 subjects were randomly assigned to each group (BRD, WRD, or WD). Four of them (two WRD and two WD) withdrew their consent, so the final analysis used data from 35 subjects (Fig. 1). The general information for the subjects in this study is presented in Table 2. The average age of the subjects was 21.8 ± 2.0 years, and there were no statistically significant differences in age among the groups. The anthropometric data of the subjects (weight, height, and BMI) and their blood pressure, pulse, stool frequency per week, and stool weight did not differ significantly among the groups.

**Table 2:** General characteristics of the subjects.

Variables	BRD (*n* = 13)	WRD (*n* = 13)	WD (*n* = 13)	Total (*n* = 39)	*p* value^a^
Age, years	22.6 ± 2.5	21.8 ± 2.1	21.2 ± 1.1	21.8 ± 2.0	0.182
Sex (male/female)	0/13	0/13	0/13	0/39	–
Height (cm)	161.8 ± 6.4	162.5 ± 5.3	159.1 ± 2.6	161.2 ± 5.1	0.189
Weight (kg)	53.2 ± 5.3	54.1 ± 6.6	53.1 ± 7.0	53.5 ± 6.2	0.906
Body mass index (kg/m^2^)	20.3 ± 1.7	20.5 ± 2.8	21.0 ± 2.8	20.6 ± 2.4	0.785
Systolic blood pressure (mmHg)	104.1 ± 8.4	101.2 ± 7.1	105.8 ± 11.6	103.7 ± 9.2	0.459
Diastolic blood pressure (mmHg)	64.7 ± 6.0	65.5 ± 4.8	69.2 ± 7.0	66.5 ± 6.2	0.137
Pulse (bpm)	76.8 ± 8.3	76.1 ± 8.5	77.2 ± 6.9	77.0 ± 7.9	0.895
Number of bowel movements per week	3.4 ± 1.1	2.8 ± 0.9	2.8 ± 0.9	3.0 ± 1.0	0.241
Stool weight (g)	135.6 ± 89.1	112.8 ± 124.7	96.0 ± 65.6	114.8 ± 95.1	0.578

Data are presented as mean ± SD.

*BRD* brown rice-based diet, *WRD* white rice-based diet, *WD* wheat-based diet, *bpm* beats per minute.

^a^Analyzed by one-way analysis of variance.

### Change in CTT

The BRD group showed a statistically significant decrease in the CCT of the left colon (*p* = 0.030) and the total colon (*p* = 0.032) after the four-week intervention (Table 3). Although the three groups did not differ in the transit time of the right colon, the left colon, and the rectosigmoid colon, the TCTT did differ significantly (*p* = 0.028) among the three groups. The TCTTs of the BRD and WD groups decreased significantly compared with that of the WRD group (*p* = 0.028 and *p* = 0.022, respectively).

**Table 3:** Changes in the colonic transit time, short-chain fatty acid content, and enzyme activity in the feces of the subjects.

	BRD (*n* = 13)			WRD (*n* = 13)			WD (*n* = 13)			*P* value^b^
	Week 0	Week 4	*p* value^a^	Week 0	Week 4	*p* value^a^	Week 0	Week 4	*p* value^a^	
Right CTT (hours)	8.9 ± 8.2	6.2 ± 6.3	0.207	12.0 ± 12.9	11.7 ± 9.2	0.831	12.8 ± 12.3	4.4 ± 3.3	0.042	0.224
Left CTT (hours)	15.4 ± 14.9	8.7 ± 8.3	0.030	12.1 ± 13.2	17.6 ± 14.1	0.274	10.3 ± 11.3	5.9 ± 7.4	0.203	0.052
Rectal CTT (hours)	18.7 ± 18.8	11.5 ± 13.8	0.204	12.2 ± 9.7	13.0 ± 10.4	0.280	15.4 ± 21.5	11.2 ± 14.1	0.600	0.525
Total CTT (hours)	42.9 ± 26.0	26.4 ± 17.9	0.032	35.4 ± 26.0	42.2 ± 23.9	0.166	38.6 ± 29.0	21.5 ± 17.1	0.063	0.028^**c**^
Stool frequency (weekly)	3.4 ± 1.1	5.0 ± 1.1	0.002	2.8 ± 0.8	4.2 ± 1.1	0.001	2.8 ± 1.0	3.9 ± 1.3	0.012	0.520
Stool weight (g)	135.6 ± 89.1	151.8 ± 69.1	0.421	112.8 ± 124.7	103.2 ± 42.7	0.842	95.9 ± 65.6	97.2 ± 47.6	0.848	0.803
Fecal lactic acid (ppm)	5.3 ± 7.9	1.0 ± 1.0	0.080	5.3 ± 6.3	0.5 ± 0.3	0.028	13.2 ± 13.4	1.9 ± 2.9	0.031	0.221
Fecal butyric acid (ppm)	33.2 ± 14.9	33.2 ± 13.2	0.988	32.2 ± 9.0	22.4 ± 13.4	0.079	33.1 ± 9.9	31.8 ± 8.0	0.636	0.272
Fecal propionic acid (ppm)	24.0 ± 14.7	20.0 ± 9.1	0.446	22.2 ± 10.9	12.1 ± 7.6	0.020	26.1 ± 13.6	21.8 ± 8.6	0.035	0.519
Fecal β-glucosidase (U/L)	111.5 ± 92.1	105.5 ± 90.5	0.736	318.3 ± 313.0	250.9 ± 211.9	0.393	168.5 ± 129.0	100.2 ± 73.9	0.042	0.515
Fecal urease (U/L)	67.3 ± 26.6	44.4 ± 23.3	0.015	82.5 ± 17.4	53.7 ± 23.5	0.006	84.0 ± 17.5	61.3 ± 24.0	0.006	0.808
Fecal β-glucuronidase (U/mL)	0.2 ± 0.1	0.2 ± 0.1	0.057	0.3 ± 0.4	0.2 ± 0.1	0.374	0.3 ± 0.3	0.5 ± 1.1	0.518	0.450

Data are presented as mean ± SD.

*BRD* brown rice-based diet, *WRD* white rice-based diet, *WD* wheat-based diet, *CTT* colonic transit time.

^a^Analyzed by paired *t*-test; *p* value indicates significant differences within treatment groups from baseline.

^b^Analyzed by repeated measure analysis of variance; *p* value indicates significant differences between treatment groups from baseline. Boldface type indicates *p* < 0.05.

^c^Linear mixed-effect model with Bonferroni test (BRD vs. WRD *p* value = 0.028, WRD vs. WD *p* value = 0.022).

### Stool frequency and improvement in rate of bowel movements

The stool frequency of the BRD group increased significantly from 3.4 ± 1.1 times/week before the study to 5.0 ± 1.1 times/week after the study (*p* < 0.002). The stool frequency of the WD group also increased significantly, from 2.8 ± 1.0 times/week before the study to 3.9 ± 1.3 times/week after the study (*p* < 0.012). The stool frequency of the WRD group tended to increase after the study, but the stool frequency before and after the study did not differ significantly among the groups **(**Table 3**)**.

### Fecal short-chain fatty acids (SCFAs) and fecal enzyme activity

In this study, the changes in fecal SCFAs and fecal enzyme activity according to the dietary intervention groups are presented in **(**Table 3**)**. Although there was a tendency toward decreasing levels of fecal SCFAs and fecal enzyme activity after the participation compared to before the study, there was no significant difference between the groups. Although there were significant decreases in the fecal lactic acid and propionic acid of the SCFAs in the WRD and WD groups after the study compared to before the study (Week 0) (*p* < 0.05), there was no significant difference before and after the study in the BRD group. Fecal enzyme activity (β-glucosidase, β-glucuronidase, and urease) showed no significant differences among dietary intervention groups (*p* > 0.05). The urease level, however, was significantly reduced after the participation compared to before the study (Week 0) in all dietary intervention groups (*p* < 0.05). In particular, in the WD group, both the fecal β-glucosidase and urease level decreased significantly after the participation compared to before the study (Week 0) (*p* < 0.05).

### Adverse events and safety monitoring

The diagnostic examination results of the subjects are presented in Supplementary Table 1, available in an online appendix. We found no subjective or objective adverse reactions or clinically meaningful changes in the physical examinations, vital signs, or diagnostic examinations. In other words, the average serum liver function, blood glucose levels, blood glucose formation, and blood biochemical indicators all stayed within the normal ranges in subjects consuming the BRD, WRD, and WD diets, and none of those indicators changed during the clinical intervention period. Therefore, all three diets were adequate and safe.

### Adherence to test diets

This study was conducted by monitoring the dietary intake of the subjects every day for four weeks (a total of 28 days) during the study period, during which the total number of uneaten meals during the study period should not exceed 25 meals per subject compared to the total of 84 meals. The dietary compliance with the test diet was more than 70% for all subjects.

### Dietary intake during the period of dietary intervention

The average dietary intake during the four-week intervention period is presented in Table 4. The WD group had a higher calorie intake level than the other groups, but that difference was not significant. Although the overall carbohydrate intake did not differ significantly among the groups, the carbohydrate intake ratios for the BRD and WRD groups were 62.0% and 63.6%, respectively, compared with 54.3% in the WD group, which was a significant difference (*p* < 0.0001). The overall protein intake (*p* < 0.0001) and protein intake percent of the rice-based (BRD and WRD) groups were significantly higher than in the WD group (*p* < 0.0001). The overall lipid intake (*p* < 0.0001) and lipid intake ratio of the WD group were significantly higher than those of the rice-based (BRD and WRD) groups (*p* < 0.0001). Also, the intake of saturated fatty acids in the WD group was significantly higher than that in the rice-based (BRD and WRD) groups (*p* < 0.0001). The daily dietary fiber intake was the highest in the BRD group, which consumed 21.5 ± 2.3 g/day, whereas the WRD and WD groups consumed 16.7 ± 4.7 g and 14.2 ± 4.5 g, respectively, which was a significant difference (*p* < 0.0002). The BRD group consumed significantly more vitamin A (*p* < 0.0001), β-carotene (*p* < 0.0001), vitamin D (*p* < 0.0001), vitamin E (*p* < 0.007), vitamin K (*p* < 0.0001), vitamin C (*p* < 0.043), vitamin B_6_ (*p* < 0.006), folate (*p* < 0.0001), K (*p* < 0.0001), Zn (*p* < 0.0001), and iron (*p* < 0.008) than the WD group.

**Table 4:** Nutrient intake by the subjects during the intervention period.

Nutrients	BRD (*n* = 13)	WRD (*n* = 13)	WD (*n* = 13)	*P* value^*^
Energy (kcal)	1434.4 ± 134.5	1410.3 ± 222.4	1597.8 ± 322.8	0.128
Carbohydrates (g)	222.5 ± 25.1	223.9 ± 35.9	295.0 ± 20.3	0.252
Total carbohydrates (%)	62.0 ± 2.9^a^	63.6 ± 4.4^a^	54.3 ± 13.0^b^	<0.0001
Total protein (%)	18.4 ± 1.2^a^	17.6 ± 2.1^a^	12.4 ± 0.3^b^	<0.0001
Total lipids (%)	22.5 ± 2.1^a^	19.0 ± 3.3^b^	36.4 ± 1.7^b^	<0.0001
Total lipids (g)	35.8 ± 4.1^b^	30.0 ± 7.2^b^	65.0 ± 18.7^a^	<0.0001
Plant lipids (g)	21.2 ± 2.4^b^	15.4 ± 3.8^b^	37.9 ± 12.1^a^	<0.0001
Animal lipids (g)	14.6 ± 2.1^b^	14.6 ± 3.6^b^	25.8 ± 6.3^a^	<0.0001
Protein (g)	65.9 ± 6.4^a^	62.2 ± 12.9^a^	50.0 ± 12.4^b^	0.002
Plant protein (g)	32.5 ± 2.9	27.6 ± 5.4	28.2 ± 7.6	0.074
Animal protein (g)	33.4 ± 4.5^a^	34.6 ± 8.2^a^	22.2 ± 6.4^b^	<0.0001
Fiber (g)	21.5 ± 2.3^a^	16.7 ± 4.7^b^	14.2 ± 4.5^b^	0.0002
Vitamin A (µg RE)	935.1 ± 146.9^a^	896.3 ± 251.3^a^	608.0 ± 146.7^b^	<0.0001
β-Carotene (µg)	5098.7 ± 814.3^a^	4829.8 ± 1452.6^a^	1972.0 ± 776.3^b^	<0.0001
Vitamin D (mg α-TE)	5.6 ± 1.0^a^	4.6 ± 1.1^b^	1.9 ± 0.4^c^	<0.0001
Vitamin E (mg)	17.6 ± 2.2^a^	15.6 ± 3.6^ba^	13.4 ± 3.4^b^	0.007
Vitamin K (µg)	338.2 ± 47.8^a^	283.9 ± 101.3^a^	113.9 ± 35.7^b^	<0.0001
Vitamin C (mg)	94.7 ± 12.7	87.7 ± 26.7	76.1 ± 19.8	0.043
Vitamin B_6_ (mg)	2.1 ± 0.2^a^	1.5 ± 0.3^b^	1.8 ± 0.8^ba^	0.006
Folate (µg)	488.9 ± 55.3^a^	463.7 ± 122.7^a^	315.9 ± 83.4^b^	<0.0001
Vitamin B_1_ (mg)	7.9 ± 1.6	6.9 ± 1.8	6.4 ± 2.4	0.167
Vitamin B_2_ (mg)	1.0 ± 0.1	0.9 ± 0.2	1.1 ± 0.3	0.176
Ca (mg)	372.4 ± 54.4	325.0 ± 98.3	343.0 ± 81.1	0.340
Na (mg)	4201.7 ± 668.7	4107.4 ± 1397.7	5284.9 ± 6437.4	0.707
K (mg)	2857.9 ± 276.5^a^	2450.8 ± 649.4^a^	1494.9 ± 388.0^b^	<0.0001
Mg (mg)	89.8 ± 13.1	86.4 ± 19.9	83.5 ± 92.7	0.9582
Zn (mg)	9.7 ± 0.8^a^	9.2 ± 1.8^a^	6.2 ± 1.3^b^	<0.0001
Iron (mg)	11.8 ± 1.2^a^	12.3 ± 2.7^a^	9.5 ± 2.5^b^	0.008
Cholesterol (mg)	279.3 ± 43.6	285.8 ± 57.3	384.7 ± 215.2	0.101
Total fatty acids (g)	23.1 ± 3.8	22.6 ± 5.2	60.6 ± 82.0	0.096
Saturated fatty acids (g)	6.9 ± 1.4^a^	7.4 ± 1.6^b^	19.6 ± 4.7^b^	<0.0001

Data are presented as mean ± SD.

^a–c^Mean with different superscripts in a row are significantly different at *p* < 0.05 by post-hoc Bonferroni multiple range test.

*BRD* brown rice-based diet, *WRD* white rice-based diet, *WD* wheat-based diet.

*Analyzed by one-way analysis of variance between groups.

## Discussion

This pilot study was conducted to evaluate whether rice-based diets (BRD and WRD) and a wheat-based diet (WD) improved the bowel health of young women with functional constipation. The clinical treatment for chronic constipation generally involves increased dietary fiber intake, volumetric relief, magnesium chloride, non-absorbable polysaccharides, probiotics, and increased body activity [3]. The major food that causes constipation is refined grains, which have a low dietary fiber level, and a diet biased toward such foods can lead to a shortage of dietary fiber and thus to constipation. In general, improved bowel function is defined as reduced CTT, increasingly regular bowel movements, or an increase in the number of bowel movements. We verified that the BRD group saw significant decreases in the left CTT (−6.7 h) and TCTT (−16.5 h), as well as an increase in the number of bowel movements after the four-week study compared to the baseline. The TCTT of the BRD group also decreased significantly compared with that of the WRD group. Thus, the BRD improved bowel function more effectively than the WRD. The TCTT of the WD group also showed a significant decrease compared with the TCTT of the WRD group, confirming that the WD was superior to the WRD in terms of improving CTT. Thus, the BRD and WD both improved bowel function more effectively than the WRD, which was based on a refined grain (*p* = 0.028). The difference between the BRD and WRD was the type of rice (the dietary fiber in brown rice was +5 g/day vs. that in white rice). Even though the daily intake of energy and nutrients did not differ between the two groups, the BRD group showed significantly improved TCTT compared with the WRD group. The BRD group naturally consumed an increased amount of insoluble dietary fiber, which facilitated bowel movements, improved bowel function, softened bowel movements, and thereby reduced constipation. Woo et al. [29] showed that constipation patients who consumed a vegetable powder containing chicory, broccoli, and whole grains had increased bowel movement frequency compared with those who consumed a dietary fiber supplement produced from a single ingredient [23, 29]. In this study, the WRD group consumed mainly soluble dietary fiber derived from white rice and side dishes, and the BRD group consumed mixed dietary fiber, the insoluble dietary fiber in the brown rice and the soluble fiber in the side dishes. We attribute the positive changes in bowel function seen in the BRD group to the mixed dietary fiber meal components. However, a more detailed analysis should be conducted in the future to verify that conclusion.

Because this study was designed to improve bowel function, all subjects were offered a diet containing 30 g of dietary fiber per day. However, the actual daily intake of dietary fiber by the study subjects differed from that significantly during the study period. The daily intake of dietary fiber in the BRD group was 21.5 ± 2.3 g/d, which is higher than the daily intake of fiber recommended for Koreans (20 g/d) [26], but the intake levels of the WRD and WD groups were only 16.7 ± 4.7 g/d and 14.2 ± 4.5 g/d, respectively, both lower than the daily intake of fiber recommended for Koreans. It is important to note that in this study, the WD group consumed 2 g less dietary fiber per day than the WRD group, but the TCTT of the WD group was superior to that of the WRD group. According to Lawton et al. [30], wheat bran fibers are more resistant to fermenting and water binding (1 g of fiber binds about 3 g of water) than oats and barley, which increases stool bulking and promotes bowel movements [31]. We attribute the greater decrease in TCTT in the WD group compared with the WRD group to the non-solubility of wheat bran fibers and their slow fermentation in the bowels. Therefore, the factors that affect bowel function have different effects depending on the type of fiber or the level of fermentation by intestinal microorganisms, in addition to the amount of dietary fiber. Based on a systematic review of 65 intervention studies of cereal fibers, Jan [16] reported that among those with 48 h or more of initial transit time, the TCCT could be decreased by 0.78 ± 0.13 h by applying 1 g/day of wheat dietary fiber. To improve bowel function effectively, dietary fiber must have a moderate degree of water solubility and ferment slowly in the colon [4, 32]. Among healthy people eating North American food, an increase in soluble and insoluble dietary fiber from cereals increased the number of bowel movements and reduced the CTT better than cereals containing only insoluble fiber [23]. In this study, the number of bowel movements in the BRD group increased by 1.6 ± 1.1 movements/week to 5.0 ± 1.1 movements/week, compared with 3.4 ± 1.1 movements/week before the study. Thus, after the study, the bowel function of the BRD group was in the normal range (more than 4 movements/week). The number of bowel movements in the WD group also increased significantly (by 1.1 ± 1.1 movements/week) to 3.9 ± 1.3 movements/week, compared with 2.8 ± 1.0 movements/week before the study. Therefore, the number of bowel movements in the BRD and WD groups increased significantly compared with the WRD group, indicating their superior effectiveness in improving bowel function. Although the BRD group was not superior to the WD group functionally, the BRD group improved TCCT and the number of bowel movements compared with the WRD group, whereas the WD group was superior to the WRD group only in improving CCT.

After subjects participated in the dietary intervention study for four weeks, there were no significant changes in SCFAs, but the fecal enzyme activity tended to decrease. In general, it has been established that increases in the fecal enzyme activities of β-glucuronidase, β-glucosidase, and urease produce mutagens or carcinogens and become colon cancer risk markers and to be highly relevant to the incidence of colorectal cancer [33]. Grasten et al. [23] showed that the intake of whole-meal rye breads reduced CTT compared to wheat breads and decreased β-glucosidase and β–glucuronidase levels due to the dilution effect of colon contents through the intake of abundant dietary fiber [23]. Also, they assumed that the intake of oatmeal porridge of 60 g/d [34] contributed to the decrease in the favorable gut microflora action and the fecal urease and fecal ammonia levels due to the effects of prebiotics. In this study, however, there was a slight change in the enzymes in the BRD group, but there was a significant decrease in the fecal enzyme (β-glucosidase and urease) activity in the WD group, even though the WD group consumed 14.2 g of dietary fiber per day during the four weeks of the study, which is very small compared to the intake of 21.5 g/d in the BRD group. Unlike the other prior reports that the effects of the fecal enzyme activity were caused by high fiber intake or prebiotics effects, it is assumed that the reduction factor in the WD group is probably due to a decrease in the intake of animal proteins. Based on Choi and Ha [35], the animal dietary intake makes the enzyme activities of β-glucosidase, β-glucuronidae, and urease 1.8~2.0 times higher than the plant dietary intake, and the enzyme activity of intestinal bacteria varies significantly depending on the dietary type. That is, the animal protein dietary intake raises the intestinal pH through the production of ammonia and acts as an unfavorable gut microflora. Also, it increases the fecal enzyme activity (β-glucuronidase, β-glucosidase, and urease), and it can be a factor in increasing colon cancer due to the increase of secondary bile acid or indol [36, 37]. Therefore, it potentially suggests that the dietary intake for the WD group may work in favor of reducing risk indicator levels for colon cancer, but further studies are needed on the impact and relevance of intestinal microflora functions.

The strengths of this study are as follows. First, we strictly monitored dietary intake by directly providing all meals to our subjects for four weeks and asking them to complete accurate dietary intake surveys. Second, we found objectively that consuming whole grains is superior to consuming refined grains in treating functional constipation by comparing and evaluating the effects of grains (rice vs. wheat) consumed in daily life rather than considering the intake of single foods. However, this study also has several limitations. First, the amount of dietary intake before the study was not investigated for all subjects, so it was not possible to compare the dietary intakes before the study period. Second, the number of people who participated in this study was somewhat small, which could limit the generalizability of the results. Third, we did not show that a rice-based diet is significantly more effective in improving intestinal health and bowel function than a wheat-based diet, so we recommend performing a large-scale clinical study.

## Conclusion

The brown rice-based and wheat-based diets used in this study for four weeks effectively improved bowel function by significantly decreasing colonic transit time and increasing the number of bowel movements compared with the group that ingested the white rice-based diet. It was not possible to show that the brown rice-based diet was functionally superior to the wheat-based diet.

## Supplementary information

Supplementary files

## Data Availability

The datasets generated and/or analyzed during the current study are not publicly available to protect patient confidentiality but are available from the corresponding author on reasonable request.

## References

[1] Guerin A, Mody R, Fok B, Lasch KL, Zhou Z, Wu EQ (2014). Risk of developing colorectal cancer and benign colorectal neoplasm in patients with chronic constipation. Aliment Pharm Ther.

[2] Kim J, Kim O, Yoo H, Kim T, Kim W, Yoon Y (2006). Effects of fiber supplements on functional constipation. Korean J Nutr.

[3] Shin JE, Jung HK, Lee TH, Jo Y, Lee H, Song KH (2016). Guidelines for the diagnosis and treatment of chronic functional constipation in Korea, 2015 Revised Edition. J Neurogastroenterol Motil.

[4] Eswaran S, Muir J, Chey WD (2013). Fiber and functional gastrointestinal disorders. Am J Gastroenterol.

[5] Gwee KA, Ghoshal UC, Gonlachanvit S, Chua AS, Myung SJ, Rajindrajith S (2013). Primary care management of chronic constipation in Asia: the ANMA chronic constipation tool. J Neurogastroenterol Motil.

[6] Yang J, Wang HP, Zhou L, Xu CF (2012). Effect of dietary fiber on constipation: a meta analysis. World J Gastroenterol.

[7] Lambeau KV, McRorie JW (2017). Fiber supplements and clinically proven health benefits: How to recognize and recommend an effective fiber therapy. J Am Assoc Nurse Pr.

[8] Watanabe N, Suzuki M, Yamaguchi Y, Egashira Y (2018). Effects of resistant maltodextrin on bowel movements: a systematic review and meta-analysis. Clin Exp Gastroenterol.

[9] Chen HL, Cheng HC, Wu WT, Liu YJ, Liu SY (2008). Supplementation of konjac glucomannan into a low-fiber Chinese diet promoted bowel movement and improved colonic ecology in constipated adults: a placebo-controlled, diet-controlled trial. J Am Coll Nutr.

[10] Micka A, Siepelmeyer A, Holz A, Theis S, Schon C (2017). Effect of consumption of chicory inulin on bowel function in healthy subjects with constipation: a randomized, double-blind, placebo-controlled trial. Int J Food Sci Nutr.

[11] McRorie JW (2015). Evidence-based approach to fiber supplements and clinically meaningful health benefits, part 1: what to look for and how to recommend an effective fiber therapy. Nutr Today.

[12] McRorie JW (2015). Evidence-based approach to fiber supplements and clinically meaningful health benefits, part 2: what to look for and how to recommend an effective fiber therapy. Nutr Today.

[13] Ansell J, Butts CA, Paturi G, Eady SL, Wallace AJ, Hedderley D (2015). Kiwifruit-derived supplements increase stool frequency in healthy adults: a randomized, double-blind, placebo-controlled study. Nutr Res.

[14] Alexandre V, Bertin C, Boubaya M, Airinei G, Bouchoucha M, Benamouzig R (2016). Randomized clinical trial: efficacy of a food supplement, TRANSITECH, on healthy individuals with mild intermittent constipation. Eur J Gastroenterol Hepatol.

[15] McRorie JW, Chey WD (2016). Fermented fiber supplements are no better than placebo for a laxative effect. Dig Dis Sci.

[16] de Vries J, Miller PE, Verbeke K (2015). Effects of cereal fiber on bowel function: a systematic review of intervention trials. World J Gastroenterol.

[17] Lembo A, Camilleri M (2003). Chronic constipation. N. Engl J Med.

[18] Schiller LR (2001). Review article: the therapy of constipation. Aliment Pharm Ther.

[19] Vuholm S, Nielsen DS, Iversen KN, Suhr J, Westermann P, Krych L (2017). Whole-grain rye and wheat affect some markers of gut health without altering the fecal microbiota in healthy overweight adults: a 6-week randomized trial. J Nutr.

[20] Holma R, Hongisto SM, Saxelin M, Korpela R (2010). Constipation is relieved more by rye bread than wheat bread or laxatives without increased adverse gastrointestinal effects. J Nutr.

[21] Jenkins DJ, Kendall CW, Vuksan V, Augustin LS, Li YM, Lee B (1999). The effect of wheat bran particle size on laxation and colonic fermentation. J Am Coll Nutr.

[22] Taniguchi K, Komae K, Takahashi A, Yoshioka T, Sone Y (2017). Effect of waxy barley, Kirarimochi, consumption on bowel movements of late-stage elderly residents at Roken nursing home. J Physiol Anthropol.

[23] Grasten SM, Juntunen KS, Poutanen KS, Gylling HK, Miettinen TA, Mykkanen HM (2000). Rye bread improves bowel function and decreases the concentrations of some compounds that are putative colon cancer risk markers in middle-aged women and men. J Nutr.

[24] Vuksan V, Jenkins AL, Jenkins DJ, Rogovik AL, Sievenpiper JL, Jovanovski E (2008). Using cereal to increase dietary fiber intake to the recommended level and the effect of fiber on bowel function in healthy persons consuming North American diets. Am J Clin Nutr.

[25] Longstreth GF, Thompson WG, Chey WD, Houghton LA, Mearin F, Spiller RC (2006). Functional bowel disorders. Gastroenterology.

[26] The Korean Nutrition Society. Dietary Reference Intakes for Koreans (KDRIs) 2010. Seoul: The Korean Nutrition Society, 2010.

[27] Metcalf AM, Phillips SF, Zinsmeister AR, MacCarty RL, Beart RW, Wolff BG (1987). Simplified assessment of segmental colonic transit. Gastroenterology.

[28] Arhan P, Devroede G, Jehannin B, Lanza M, Faverdin C, Dornic C (1981). Segmental colonic transit time. Dis Colon Rectum.

[29] Woo HI, Kwak SH, Lee Y, Choi JH, Cho YM, Om AS (2015). A controlled, randomized, double-blind trial to evaluate the effect of vegetables and whole grain powder that is rich in dietary fibers on bowel functions and defecation in constipated young adults. J Cancer Prev.

[30] Lawton CL, Walton J, Hoyland A, Howarth E, Allan P, Chesters D (2013). Short term (14 days) consumption of insoluble wheat bran fibre-containing breakfast cereals improves subjective digestive feelings, general wellbeing and bowel function in a dose dependent manner. Nutrients.

[31] Cummings J. The effect of dietary fiber on fecal weight and composition. In: Spiller GS, editor. CRC Handbook on dietary fiber in nutrition. 2nd ed. Boca Raton, FL: CRC Press; 1993.

[32] lee J, Kim D, Yoon I, Jung K (2016). Diet and nutritional management in functional gastrointestinal disorder constipation. Korean J Med.

[33] Goldin BR (1990). Intestinal microflora: metabolism of drugs and carcinogens. Ann Med.

[34] Valeur J, Puaschitz NG, Midtvedt T, Berstad A (2016). Oatmeal porridge: impact on microflora-associated characteristics in healthy subjects.. Br J Nutr..

[35] Choi SS, Ha NJ (1999). Fecal microflora of mice in relation to diet. Kor J Micro.

[36] Rowland IR. Toxicology of the colon. In: Gibson GR, Macfarlane GT editors. Human Colonic Bacteria: Role of the intestinal microflora. Boca Raton, FL: CRC Press; 1995. p. 155–74.

[37] Clinton SK, Bostwick DG, Olson LM, Mangian HJ, Visek WJ (1988). Effects of ammonium acetate and sodium cholate on N-methyl-N'-nitro-N-nitrosoguanidine-induced colon carcinogenesis of rats. Cancer Res..

