# Supplementary material for: Effects of rice-based and wheat-based diets on bowel movements in young Korean women with functional constipation
Source: Eur J Clin Nutr. 2020 Apr 22;74(11):1565–75. doi: 10.1038/s41430-020-0636-1 (PMC7606135; doi:10.1038/s41430-020-0636-1)
Supplement: Supplementary file 1 — Supplementary files [file 41430_2020_636_MOESM1_ESM.docx]

**Supplementary files list**

**Supplementary Figure 1.** Test meals in this study (examples)

| **Meals** | **Brown rice-based diet (BRD)** | **White rice-based diet (WRD)** | **Wheat-based diet**  **(WD)** |
| --- | --- | --- | --- |
| **Break**  **-fast** | 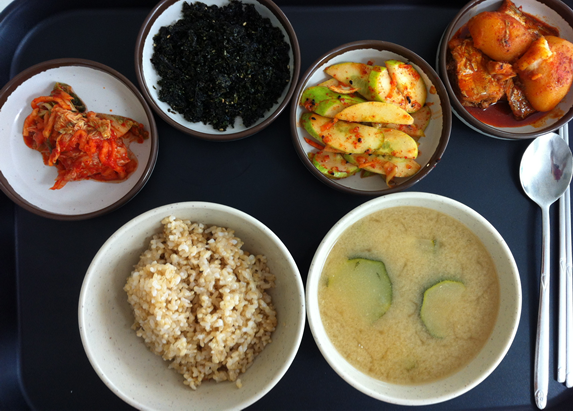 | 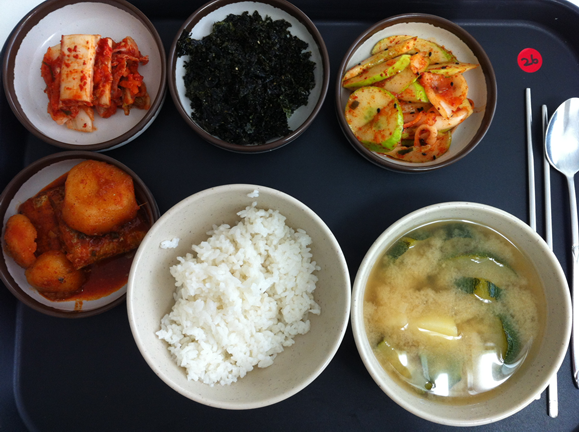 | 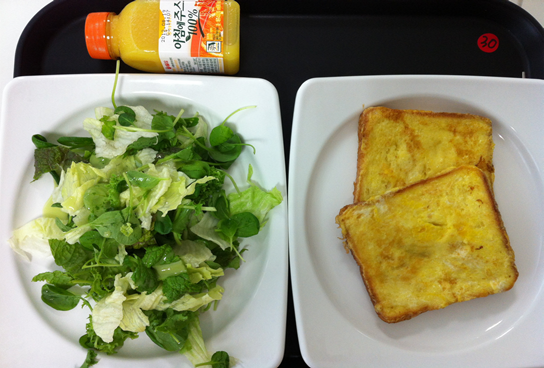 |
|  | • Brown rice | • White rice | • Egg toast |
|  | • Deonjang (fermented soy) soup | • Deonjang  (fermented soy) soup | • Vegetable salad |
|  | • Kimchi | • Kimchi | • Orange juice |
|  | • Cucumber salad | • Cucumber salad |  |
|  | • Seasoned laver | • Seasoned laver |  |
|  | • Braised  cutlassfish (mackerel) | • Braised  cutlassfish (mackerel) |  |
| **Lunch** | 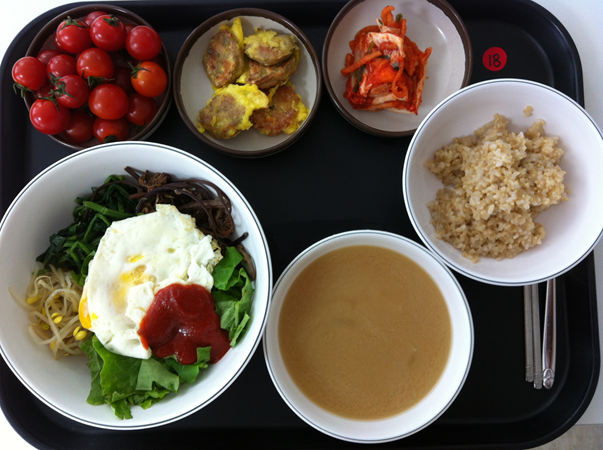 | 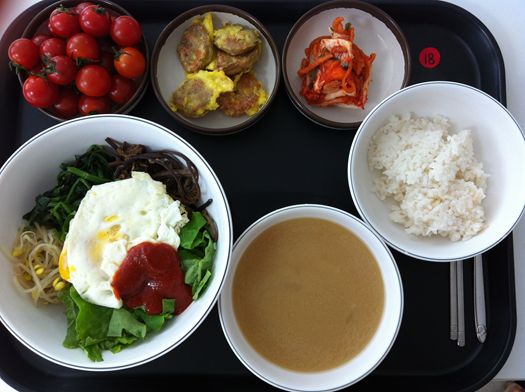 | 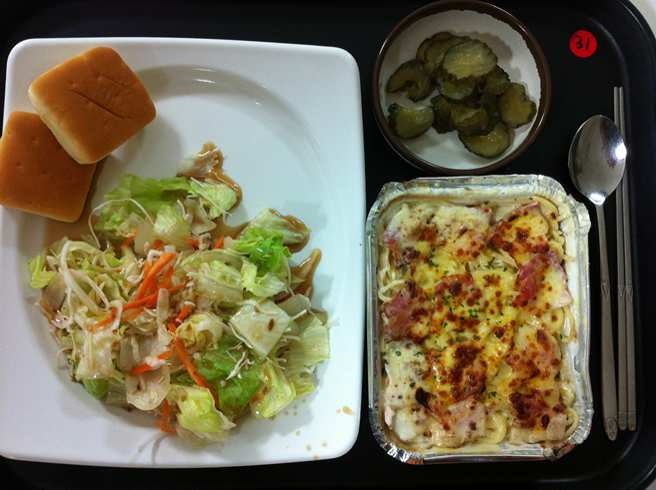 |
|  | • Bibimbap (brown rice) | • Bibimbap (white rice) | • Cream spaghetti |
|  | • Deonjang  (fermented soy) soup | • Deonjang  (fermented soy) soup | • Vegetable/fruit salad |
|  | • Kimchi | • Kimchi | • Pickled cucumber |
|  | • Cherry tomatoes | • Cherry tomatoes | • Bread |
|  | • Pan-fried beef meatballs | • Pan-fried beef meatballs |  |
| **Dinner** | 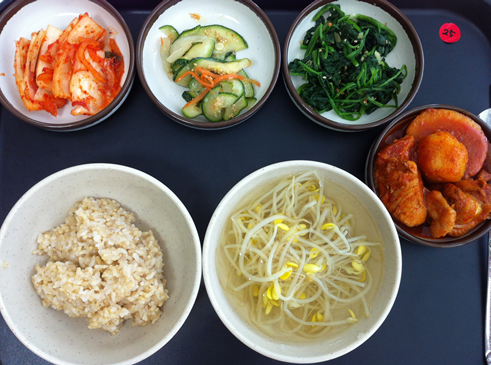 | 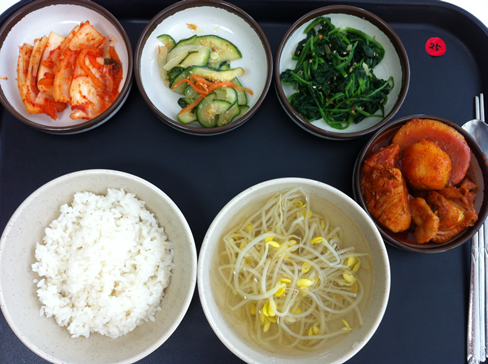 | 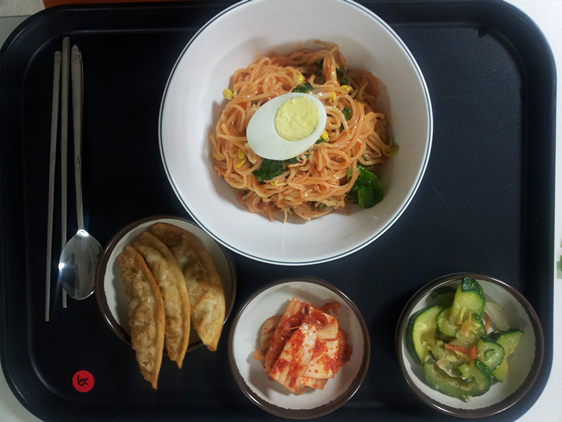 |
|  | • Brown rice | • White rice | • Spicy noodles |
|  | • Bean sprout soup | • Bean sprout soup | • Fried dumplings |
|  | • Kimchi | • Kimchi | • Stir-fried pumpkin |
|  | • Stir-fried pumpkin | • Stir-fried pumpkin | • Kimchi |
|  | • Seasoned spinach | • Seasoned spinach |  |
|  | • Stir-fried pork  with Korean chili peppers | • Stir-fried pork  with Korean chili peppers |  |

| **Supplementary figure 2.** Representative abdominal X-rays data at baseline and 4 week  in a subjects(example) | |
| --- | --- |
| **BRD group (Age 22 , Female)** | |
| **Before (baseline)** | **After (4 week)** |
|  |  |
| Number of markers present: 57  Colon transit time(h): 68.4 | Number of markers present: 22  Colon transit time(h): 26.4 |
| **WRD group (Age 21, Female)** | |
|  |  |
| Number of markers present: 60  Colon transit time(h): 72.0 | Number of markers present: 44  Colon transit time(h): 52.8 |
| **WD group (Age 22, Female)** | |
|  |  |
| Number of markers present: 43  Colon transit time(h): 51.6 | Number of markers present: 22  Colon transit time(h): 26.4 |

| **Supplementary Table 1.** Laboratory profiles of the participants in this study | | | | | | | |
| --- | --- | --- | --- | --- | --- | --- | --- |
| **Laboratory profiles (standard range)** | **BRD (n=13)** | | **WRD (n=13)** | | **WD (n=13)** | |  |
|  | **Week 0** | **Week 4** | **Week 0** | **Week 4** | **Week 0** | **Week 4** | **P-value^1^** |
| WBC (4.8–10.8×10^3^ /μL) | 5.58±1.05 | 5.89±0.87 | 5.81±1.35 | 6.17±1.51 | 5.48±0.88 | 6.50±1.11 | 0.248 |
| RBC (4.2–5.4×100^3^ /μL) | 4.15±0.28 | 4.18±0.28 | 4.35±0.30 | 4.39±0.34 | 4.55±0.27 | 4.59±0.22 | 0.994 |
| Hemoglobin (12–16 g/dL) | 12.40±0.88 | 12.55±1.01 | 13.28±0.96 | 13.35±1.02 | 13.16±1.13 | 13.25±0.78 | 0.831 |
| Hematocrit (37–47%) | 37.82±2.25 | 38.27±2.49 | 40.12±2.92 | 39.99±3.12 | 40.43±2.97 | 40.38±1.72 | 0.598 |
| Platelet (130–450×10^3^ /μL) | 265.31±44.71 | 267.15±38.77 | 258.85±70.74 | 266.09±60.03 | 285.46±53.77 | 315.73±56.25 | 0.076 |
| GGT (8–48 IU/L) | 12.92±5.06 | 11.85±4.38 | 11.92±3.30 | 11.45±3.86 | 13.31±5.66 | 13.55±5.48 | 0.485 |
| AST (12–33 IU/L) | 17.00±3.74 | 17.54±3.62 | 18.54±3.86 | 17.00±2.79 | 19.15±4.74 | 17.73±2.57 | 0.246 |
| ALT (5–35 IU/L) | 11.62±3.38 | 12.08±4.15 | 11.85±4.60 | 11.82±3.92 | 13.38±4.50 | 13.09±3.05 | 0.916 |
| Total bilirubin (0.2–1.2 mg/dL) | 0.95±0.37 | 0.85±0.25 | 0.88±0.33 | 0.76±0.34 | 0.73±0.24 | 0.65±0.17 | 0.946 |
| Total protein (6.7–8.3 g/dL) | 7.38±0.31 | 7.45±0.37 | 7.43±0.25 | 7.33±0.22 | 7.50±0.36 | 7.55±0.36 | 0.332 |
| Albumin (3.5–5.3 g/dL) | 4.46±0.13 | 4.55±0.18 | 4.55±0.19 | 4.50±0.17 | 4.61±0.26 | 4.65±0.22 | 0.144 |
| BUN (8–23 mg/dL) | 11.00±2.38 | 10.46±1.61 | 10.85±2.70 | 11.09±2.77 | 11.62±2.75 | 12.00±2.37 | 0.525 |
| Creatinine (0.7–1.7 mg/dL) | 0.66±0.10 | 0.64±0.07 | 0.70±0.06 | 0.65±0.09 | 0.67±0.07 | 0.62±0.10 | 0.501 |
| Glucose (74–106 mg/dL) | 76.69±6.46 | 76.85±7.07 | 80.69±7.20 | 79.73±6.40 | 81.31±7.13 | 81.64±6.22 | 0.823 |
| Total cholesterol (~200 mg/dL) | 168.00±34.54 | 160.08±33.86 | 155.38±18.86 | 151.91±17.33 | 171.23±25.06 | 164.09±22.35 | 0.614 |
| Triglyceride (~200 mg/dL) | 76.23±17.28 | 66.00±19.81 | 81.92±47.34 | 70.64±31.77 | 80.54±38.06 | 83.73±43.13 | 0.644 |
| HDL-cholesterol  (48.9–73.5 mg/dL) | 60.31±11.32 | 57.62±8.83 | 60.92±10.38 | 58.00±11.16 | 61.69±14.94 | 62.73±17.86 | 0.425 |
| LDL-cholesterol (~140 mg/dL) | 84.00±35.07 | 83.23±31.47 | 72.69±14.68 | 77.73±16.86 | 88.15±22.23 | 84.73±18.03 | 0.601 |
| Data are presented as mean ± SD.  ^1^Analyzed by repeated measure analysis of variance; p-value indicates significant differences between treatment groups from baseline.  Abbreviations: BRD, brown rice-based diet; WRD, white rice-based diet; WD, wheat-based diet | | | | | | | |
